# P04.48. Fatty acids and dementia: systematic review and meta-analyses

**DOI:** 10.1186/1472-6882-12-S1-P318

**Published:** 2012-06-12

**Authors:** M Loef, H Walach

**Affiliations:** 1 European University Viadrina, Frankfurt, Germany

## Purpose

Fatty acids are essential for neuronal functioning. Their impact on the risk of developing dementia is, however, not restricted to the intake of omega-3 polyunsaturated fatty acids (PUFAs). There is increased evidence that a diet of fats is associated with neurodegenerative diseases in a multifaceted way.

## Methods

We performed a systematic review on the association between fatty acids and dementia, Alzheimer’s disease (AD), mild cognitive impairment and cognitive decline. Studies were selected if they were systematic reviews, observational studies, or randomized controlled trials which covered the thematic overlap not detailed in earlier systematic reviews. RCT and prospective studies needed to last for at least 6 and 12 months, respectively. A meta-analysis was conducted if it was possible to pool the effect sizes of three or more studies.

## Results

Out of 5497 publications, 38 studies met the inclusion criteria and dealt with the association between dementia or cognitive decline and the total intake of fatty acids, the diet of saturated fatty acids (SFA), the diet or supplementation of ω-3 PUFAs or ω-6 PUFAs, the ratio of ω-6 to ω-3 PUFAs, and the intake of fish. Regular consumption of fish (≥ 2x/week) reduced the risk of dementia and AD by 37% and 43%, respectively. There is evidence that the increased diet or plasma levels of ω-3 and higher ω-3/ ω-6 ratios are associated with a lower risk of dementia and AD, whereas there is no consistent evidence for an effect of SFA, ω-6 PUFAs and total fatty acids on the risk of dementia.

## Conclusion

The association of dietary lipids and dementia is complex enough to hypothesize that a change of dietary patterns is more likely to lower the risk of the disease than the supplementation of a single nutrient.

